# Klippel Feil syndrome with isolated hypokinesia of the left ventricle: A rare association

**DOI:** 10.4103/0974-2069.64354

**Published:** 2010

**Authors:** Syed Ahmed Zaki, Prithi Shenoy, Preeti Shanbag

**Affiliations:** Department of Pediatrics, Lokmanya Tilak Municipal General Hospital, Sion, Mumbai, India. E-mail: drzakisyed@gmail.com

Sir,

Klippel-Feil syndrome (KFS) is defined as the congenital fusion of two or more cervical vertebrae and is believed to result from faulty segmentation along the embryo's developing axis during the second to eighth week of gestation.[1] The classical clinical triad of Klippel-Feil syndrome is low hairline, short neck, and restriction of head and neck movements. It was originally described in 1912, by Klippel and Feil, and is commonly associated with many congenital malformations.[2] We report a rare association of KFS with isolated hypokinesia of the left ventricle.

A 10-year-old boy was referred to our Out Patient Department (OPD) for restriction of neck movements. He had no other symptoms. There was no significant past history. His school performance was normal and there was no family history of heart disease. On examination he was an intelligent, prepubertal boy, with a height of 135 cm , (twenty-fifth percentile) and weight of 30 kg (fiftieth percentile,). A short neck and low occipital hairline were noted with normal facial appearance. Neck movements were restricted in both lateral and extension. No other significant findings were found on general examination. Blood pressure was 100/70 mmHg and heart sounds were normally heard. Systemic examination was normal. His hearing assessment and ophthalmological examination was normal. Chest X-ray and ultrasonography of the abdomen were normal. The lateral view of the cervical spine X-ray showed congenital fusion of the posterior segments C1-C2 and C3-C5. The X-ray of the spine was suggestive of scoliosis in the thoracic spine. A screening echocardiography that was performed showed left ventricular hypokinesia with an ejection fraction of 35%.

The importance of recognizing Klippel Feil syndrome lies in the fact that there is a strong association of this anomaly with significant abnormalities of other systems in the body. These include skeletal system abnormalities, such as, scoliosis and/or kyphosis (60%), Sprengel deformity of the scapula (30%), and torticollis, urinary system abnormalities (35%), loss of hearing (30%), facial asymmetry and flattening of neck (20%), synkinesis or mirror movements (20%), congenital heart diseases (4.2 - 14%). Brain stem anomalies, congenital cervical stenosis, adrenal aplasia, pitosis, lateral rectus muscle paralysis facial nerve paralysis, syndactilia, and diffuse or focal hypoplasia in the upper extremities may also be seen.[1] Cardiovascular anomalies have been recognized in patients with KFS with an incidence of 4.2 to 14%, as reported by several authors.[1-3] Various lesions including coarctation of the aorta occur, but ventricular septal defects are the most common.[2,3] An extensive PubMed search did not reveal even a single case of KFS associated with hypokinesia of the left ventricle. This case widens the spectrum of cardiac defects seen in this condition. Hypokinesia of the left ventricle has been described with acute myocardial infarction, systemic lupus erythematosus, and systemic scleroderma.[4,5] Our patient was asymptomatic for any of the conditions described here. Also, in view of the reported increased incidence of congenital heart lesions in the Klippel-Feil syndrome and their heterogeneity, it is probable that the association in our patient is genuine rather than fortuitous.

Through this case, we highlight the need for the cardiologist to be aware of this rare association of Klippel Feil syndrome with isolated hypokinesia of the left ventricle.
